# Δ9-THC Intoxication by Cannabidiol-Enriched Cannabis Extract in Two Children with Refractory Epilepsy: Full Remission after Switching to Purified Cannabidiol

**DOI:** 10.3389/fphar.2016.00359

**Published:** 2016-09-30

**Authors:** José A. S. Crippa, Ana C. S. Crippa, Jaime E. C. Hallak, Rocio Martín-Santos, Antonio W. Zuardi

**Affiliations:** ^1^Department of Neuroscience and Behavior, University of São PauloRibeirão Preto, Brazil; ^2^Department of Clinical Medicine, Federal University of ParanáCuritiba, Brazil; ^3^Institute of Neuroscience, Hospital Clinic, August Pi i Sunyer Biomedical Research Institute – Center for Biomedical Research in Mental Health Network and Department of Medicine – University of BarcelonaBarcelona, Spain

**Keywords:** cannabidiol, epilepsy, Cbd, intoxication, refractory reriod, electrophysiological

## Abstract

Animal studies and preliminary clinical trials have shown that cannabidiol (CBD)-enriched extracts may have beneficial effects for children with treatment-resistant epilepsy. However, these compounds are not yet registered as medicines by regulatory agencies. We describe the cases of two children with treatment-resistant epilepsy (Case A with left frontal dysplasia and Case B with Dravet Syndrome) with initial symptom improvement after the introduction of CBD extracts followed by seizure worsening after a short time. The children presented typical signs of intoxication by Δ9-THC (inappropriate laughter, ataxia, reduced attention, and eye redness) after using a CBD-enriched extract. The extract was replaced by the same dose of purified CBD with no Δ9-THC in both cases, which led to improvement in intoxication signs and seizure remission. These cases support pre-clinical and preliminary clinical evidence suggesting that CBD may be effective for some patients with epilepsy. Moreover, the cases highlight the need for randomized clinical trials using high-quality and reliable substances to ascertain the safety and efficacy of cannabinoids as medicines.

## Introduction

Novel antiepileptic compounds with new mechanisms of action, fewer side effects, and better safety and tolerability profiles have been approved over the last years. However, although more than 20 different drugs approved for the treatment of epilepsy exist today, around 30% of patients continue to have seizures (Brodie et al., 2012). Modern neurosurgery techniques are an option for these patients, but drug-resistant epileptic patients often fail to meet the clinical criteria for surgery, and a significant number of operated patients do not achieve full seizure remission. Together, these obstacles have led patients with drug-resistant epilepsy (defined as the failure to achieve seizure remission after trials with at least two adequate medications, ILAE – Blümcke et al., 2016) and their families to seek new medications, which highlights the importance of the search for novel, effective pharmacological options with improved tolerability and different mechanisms of action.

The medicinal effects of cannabis in epilepsy have been known for centuries and, nowadays, the anticonvulsant properties of its components have received increasing attention (Leo et al., 2016). The discovery of endocannabinoids and cannabinoid receptors in the brain has renewed the interest in the potential of cannabinoid compounds to treat seizures (dos Santos et al., 2015). The *Cannabis sativa* plant contains more than 400 compounds, of which 100 are known as phytocannabinoids. The two cannabinoids with the highest concentrations in cannabis are delta9-tetrahydrocannabinol (Δ9-THC), responsible for most of the psychotomimetic effects of the drug, and cannabidiol (CBD), the most common non-psychoactive cannabinoid (Crippa et al., 2010, 2015).

Anecdotal reports suggest that cannabis has an anti-seizure potential and could thus be used to treat subjects with epilepsy. Cannabis is currently approved for the treatment of epilepsy in several states of the United States and in Israel and Canada (Paolino et al., 2016; Tzadok et al., 2016). However, the lack of controlled studies and the fact that cannabis contains many other substances (including other cannabinoids at different concentrations) have hindered definitive conclusions. Moreover, reports suggest that crude cannabis may have no efficacy and may even exacerbate epileptic seizures (Tofighi and Lee, 2012; Hamerle et al., 2014).

Animal studies and preliminary clinical trials have shown significant improvements in children with treatment-resistant epilepsy treated with CBD-enriched extracts (Rosenberg et al., 2015). However, these compounds are not yet registered as medicines by regulatory agencies. The general press and social media have raised attention to these products in countries such as the United States, United Kingdom, and Brazil. This led to the development of a wide range of cannabis-derived products for oral use with no regulation, quality assurance, or accurate content labeling (Vandrey et al., 2015). Led by despair, many families have resorted to these products (usually commercialized as dietary supplements) in an attempt to control the seizures of their children (Vandrey et al., 2015). The potential chronic medical use of non-purified CBD extracts raises important concerns, particularly in children and adolescents with a developing brain. First, the long-term use of drugs containing THC may have adverse and long-lasting harmful effects such as the onset of chronic psychiatric disorders, addiction, cognitive impairment, and changes in brain function that can have an impact on educational, professional, and social achievements (Volkow et al., 2014; Andrade, 2016). Second, the use of edible products makes it difficult to titrate doses, which can lead to over- or under-dosing and implies the risk of drug-interactions with other medicines. Third, due to the necessary chronic use of these products and the long half-life of Δ9-THC, cannabis-derived products can cause intoxication in patients with clinical, cognitive, and motor impairments, with detrimental consequences (Tofighi and Lee, 2012; Hamerle et al., 2014). The intoxication effects of Δ9-THC include mild euphoria, ataxia, decreased attention, red eyes, irritability (Martin-Santos et al., 2012) and, in epilepsy patients, possible seizure worsening (Friedman and Devinsky, 2015). Here, we describe two cases of children with treatment-resistant epilepsy in seizure remission who eventually presented intoxication by Δ9-THC with the use of edible CBD-enriched extracts and who attained full remission again after switching to purified CBD.

## Case A

**FIGURE 1:**
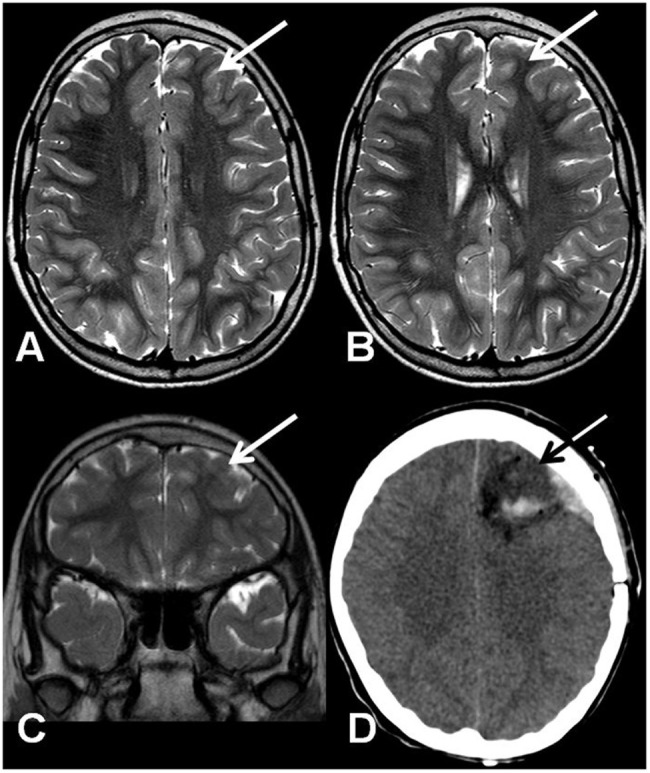
**(A,B)** Axial T2-weighted TSE MRI. Coronal T2-weighted TSE MRI **(C)** and axial computed tomography after surgery **(D)**. The arrows show a mildly hypoplastic frontal lobe with focal T2 signal hyperintensity on the cortico-subcortical transition **(A–C)** and the resected region **(D)**.

**FIGURE 2:**
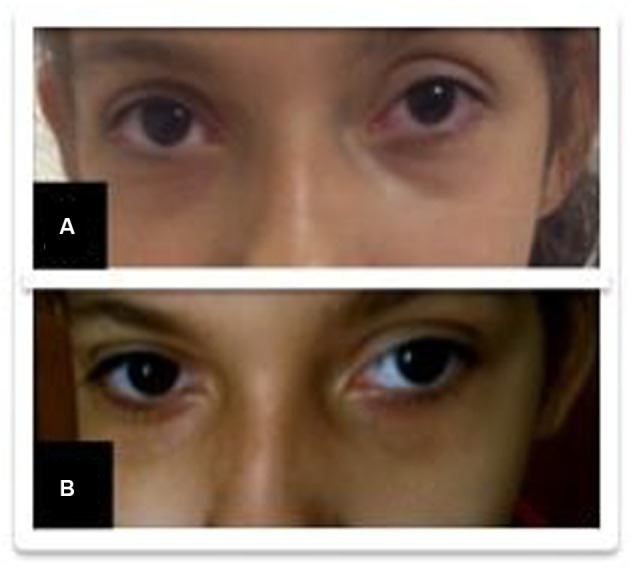
**Improvement of eye redness (from **A** to **B**) after replacement of the cannabidiol (CBD)-enriched extract**.

**FIGURE 3:**
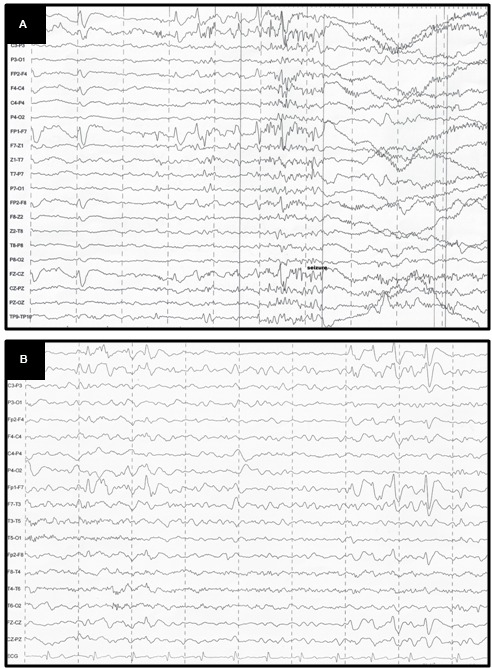
**(A)** EEG before CBD: generalized, sharp wave, regular, in burst, high voltage in the frontal areas; **(B)** EEG after CBD: diffuse slowing in the background.

A 10-year-old girl was diagnosed with refractory epilepsy and left frontal dysplasia at 5 months of age. Prior to cannabinoid treatment she presented about three complex focal seizures. Treatment with phenytoin, topiramate, carbamazepine, levetiracetam, lamotrigine, primidone, and clobazam for appropriate periods and at adequate doses did not lead to seizure remission. The patient underwent focal resection of the left anterior frontal lobe at age 8 (**Figure 1**) and was seizure-free for 4 months. Her histological diagnosis after surgery was focal cortical dysplasia type 1b. After 4 months, seizures reappeared every day in the morning, despite treatment with topiramate, valproate, and clobazam. She was then started on an oral CBD-enriched extract (16% CBD, 208 mg/day or 6 mg/Kg/day; divided in three doses of 70 mg; in addition to topiramate 1 mg/kg/day, clobazan 0.4 mg/kg/day, and valproate 12.7 mg/kg/day), which eliminated seizures and improved general behavior, speech, understanding, and attention. After 4 months, the child started to present inappropriate laughter, ataxia, reduced attention, irritability, aggressiveness, spasms, and bilateral mydriasis with eye redness (**Figure 2A**). An analysis of the extract (which remained the same during the initial treatment) detected 4.03% of Δ9-THC and 89.6% of CBD, and a hair test showed the same concentrations of the two cannabinoids. The CBD-enriched extract was then replaced by the same dose of purified CBD (99.6%, dissolved in corn oil-BSPG-Pharm, Sandwich, UK) with no Δ9-THC, which led to complete improvement of all intoxication signs (**Figure 2B**) after 1 week, as assessed by clinical evaluation and EEG results (**Figure 3**), and to complete seizure remission after 4 weeks of the new treatment.

## Case B

A 7-year-old boy had a diagnosis of refractory epilepsy due to SCN1A mutations associated with Dravet syndrome. Prior to treatment with cannabinoids, he presented daily complex, focal, myoclonic, tonic, and absence seizures. In addition to severe myoclonic epilepsy, the child also presented behavioral and developmental delays, hyperactivity and impulsiveness, and autistic-like behaviors. The boy underwent anticonvulsant treatment for appropriate periods and with adequate doses, failing to respond to several drugs including stiripentol, valproic acid, oxcarbazepine, topiramate, levetiracetam, phenytoin, phenobarbital, and sulthiame. The patient was then started on an orally administered CBD-enriched extract, 16% CBD, 250 mg/day or 12.5 mg/Kg/day; divided in two doses of 125 mg; in addition to topiramate 3.75 mg/KG/day and valproate 24 mg/kg/day), which reduced seizures (from 1–2 a day to 1 a month) and improved general behavior, speech, understanding, and attention. After 3 months, however, the patient started to present ataxia, reduced attention, irritability, aggressiveness, and seizure worsening. An analysis of the cannabis extract detected 3.1% of Δ9-THC and 91% of CBD, proportions that were also confirmed through a hair test. The CBD-enriched extracts (which remained the same during the initial treatment) was replaced by 200 mg/day of purified CBD (divided in two doses of 100mg, oral solution with 99.6% CBD dissolved in corn oil (BSPG-Pharm, Sandwich, UK) and no Δ9-THC, which was later increased to 300 mg/day divided in two doses of 150 mg. This dose led to complete improvement of all intoxication symptoms after 1 week of treatment as indicated by clinical evaluation, EEG improvements, and complete seizure remission after 3 weeks of the new treatment. The child also had a clear improvement in most of the autistic-like symptoms over the course of 6 months, including poor communication (vocabulary and spelling), poor social interaction, and repetitive and limited behavior, which allowed the boy to start school and get involved in sports activities like swimming.

Follow up assessments at 1 year (Case A) and 1 year and 10 months (Case B) showed remission of seizures and clear progressive improvement of the remaining general symptoms with the use of pure CBD. The other medications remained stable before and during the time of transition from the cannabinoid extract to the purified CBD in both cases. Likewise, there were no changes in the dose or frequency of administration of the purified CBD oil. No side-effects were reported for any dose of CBD used and plasmatic levels of the adjuvant antiepileptics did not change during the trial in the two cases.

## Discussion

The present report is in line with pre-clinical and preliminary clinical data suggesting that CBD (and perhaps Δ9-THC) may be effective for some patients with epilepsy. However, the adverse events in both children, including Δ9-THC intoxication and increased seizures, highlight the need for accurate dosing standards and quality assurance of cannabis-derived medications. The use of edible products makes it difficult to titrate doses and to establish the actual amount of cannabinoids across different products or different batches of a same product. Moreover, when taken orally, Δ9-THC leads to the synthesis of much larger amounts of 11-OH-THC compared to when it is ingested through smoking. Since 11-OH-THC is also active, it is possible that its psychopharmacological effects may combine with those of Δ9-THC to produce stronger effects in the CNS (Benjamin and Fossler, 2016). This is of particular concern when we consider that the pharmacological potency of Δ9-THC is much higher than that of CBD, and thus the amount of Δ9-THC required to produce an effect (as well as side-effects) at a given intensity is much lower than the amount of CBD. Our patients, for instance, who received 208 mg and 250 mg of CBD-enriched extract with 4.03% and 3.1% of Δ9-THC, would ingest 8.3 mg and 7.5 mg of Δ9-THC, which can be considered a very high dose (Benjamin and Fossler, 2016), especially for children.

Surveys with caregivers and patients have also examined the effects of CBD-enriched extracts in epilepsy. One of such surveys involving parents of children with severe epilepsies in a *Facebook* group reported improvements in 16 out of 19 patients treated with CBD/Δ9-THC-enriched extracts, and two patients were reported to have become seizure-free (Porter and Jacobson, 2013).

A retrospective case series of 75 children with refractory epilepsy taking oral cannabis extracts reported that 25 (33%) patients presented >50% reduction in seizure frequency, whereas 44% had adverse events including increased seizures (13%) and rare events such as developmental regression, abnormal movements, status epilepticus requiring intubation, and death (Press et al., 2015). More recently, a retrospective study described the successful effects of CBD-enriched medical cannabis in 74 children (1–18 years old) with refractory epilepsy from five Israeli pediatric epilepsy clinics (Tzadok et al., 2016). CBD-enriched treatment significantly reduced the frequency of seizures in most of the children (66/74, 89%) and only five (7%) patients reported seizure exacerbation that led to the discontinuation of treatment with CBD-enriched extracts. Despite this, minor and infrequent side effects were reported.

There are anecdotal reports describing the antiepileptic effects of crude cannabis (Friedman and Devinsky, 2015), but evidence of seizure exacerbation and lack of effects is also available (Tofighi and Lee, 2012; Hamerle et al., 2014). Since *C. sativa* contains many other substances, with different cannabinoid concentrations and ratios, there is no sufficient information to allow for definitive conclusions (Rosenberg et al., 2015). Moreover, the pharmacokinetic interactions of CBD and Δ9-THC (and of other cannabinoids) raise safety concerns, as both compounds can inhibit cytochrome P450 enzymes (CYP2C and CYP3A4) that act in the metabolism of many of the anticonvulsants commonly used with CBD (Geffrey et al., 2015).

Some therapeutic trials have tested the effects of isolated cannabinoids in the treatment of epilepsy. In an early prospective, placebo-controlled three-month trial in adults with treatment-resistant epilepsy, Mechoulam and Carlini (1978) showed that two out of four patients treated with purified CBD (200 mg/day) became seizure free and one presented partial improvement. Later, a prospective, placebo-controlled trial in adolescents and adults with treatment-resistant convulsive seizures treated for 8–18 weeks reported that four out of eight subjects in the purified CBD (200–300 mg/day) arm became seizure free (Cunha et al., 1980). More recently, in an open-label trial (Devinsky et al., 2016), patients (*N* = 162, 1–30 years-old) with severe childhood-onset refractory epilepsy were enrolled in an expanded access program. Participants were given purified oral CBD (from 2 to 5 mg/kg/day to a maximum up-titrated dose of 25 mg/kg/day) during 12 weeks. The authors reported that CBD reduced seizure frequency and presented an adequate safety profile.

The pharmacological mechanisms underlying the antiepileptic action of CBD are not yet well understood, as CBD interferes with different neurotransmitter systems in a number of ways. For instance, there is evidence that CBD inhibits the reuptake and metabolism of anandamide, increases hippocampal neurogenesis, interacts with 5HT1A and TRPV1 receptors, and presents antioxidant and neuroprotective effects which may, at least in part, help to explain its anticonvulsant profile (Reif et al., 2007; Zuardi, 2008; Campos and Guimarães, 2008; Wolf et al., 2010). Moreover, the analysis of endocannabinoid signaling elements and related proteins in lymphocytes of patients with Dravet syndrome showed changes in the alpha-1H unit of the voltage-dependent calcium channel and an up-regulation of CB2 receptors, associated with an activation of lymphocytes and changes in inflammation-related genes (Rubio et al., 2016). These changes have been described in inflammatory diseases and epilepsy and may support a potential dysregulation of the endocannabinoid system in the SNC.

We cannot exclude the possibility that the case presentations may have been impacted by the side effects and pharmacokinetic interactions of the other medications the children were taking, or even by other chemical substances that could be present in the cannabis extracts initially used. However, neither the cannabinoids nor the other medications are known to induce mydriasis or eye redness and the patients presented improvements with CBD.

The two cases reported here highlight the need for cannabis-derived compounds produced according to good laboratory and manufacturing practices (GMP/GLP) that could lead to the development of medicines consistently produced and controlled by international regulatory standards. The use of handcrafted, uncontrolled cannabis-based medicines by children and adolescents raises special concerns because of the well-known harmful long-term effects of Δ9-THC on the developing brain (Volkow et al., 2014). The potentially toxic effects of Δ9-THC and other cannabis constituents, including cognitive impairment and chronic psychiatric disturbances, have not yet been well studied in younger patients who might be more vulnerable than adults to potential long-term adverse effects (Volkow et al., 2014). Therefore, randomized clinical trials using high-quality and reliable cannabis-derived substances without other impurities (e.g., solvents) are required to ascertain the safety and efficacy of cannabinoids as medicines.

## Author Contributions

All authors had full access to all of the data in the study and take responsibility for the integrity of the data and the accuracy of the data analysis. Case study concept and design: All authors. Acquisition, analysis, or interpretation of data: All authors. Drafting of the manuscript: JC, AC, and AZ. Critical revision of the manuscript for important intellectual content: All authors. Administrative, technical, or material support: JC and AC. Study supervision: All authors.

## Conflict of Interest Statement

JH, AZ, and JC are co-inventors (Mechoulam R, JC, Guimaraes FS, AZ, JH, Breuer A) of the patent “Fluorinated CBD compounds, compositions and uses thereof. Pub. No.: WO/2014/108899. International Application No.: PCT/IL2014/050023”; Def. US no. Reg. 62193296; 29/07/2015; INPI em 19/08/2015 (BR1120150164927). University of São Paulo licensed it to Phytecs Pharm (Resolução USP No. 15.1.130002.1.1). University of São Paulo has an agreement with Prati-Donaduzzi (Toledo, Brazil): “Desenvolvimento de um produto farmacêutico contendo canabidiol sintético e comprovação de sua segurança e eficácia terapêutica na epilepsia, esquizofrenia, doença de Parkinson e transtornos de ansiedade”. JC received a travel support from BSPG-Pharm.

All the other authors declare that the research was conducted in the absence of any commercial or financial relationships that could be construed as a potential conflict of interest.
